# Long-term results of diaphragmatic plication in adults with unilateral diaphragm paralysis

**DOI:** 10.1186/1749-8090-5-111

**Published:** 2010-11-15

**Authors:** Sezai Celik, Muharrem Celik, Bulent Aydemir, Cemalettin Tunckaya, Tamer Okay, Ilgaz Dogusoy

**Affiliations:** 1 Siyami Ersek Cardiothoracic Training Hospital, Thoracic Surgery Department, Istanbul, Turkey

## Abstract

**Background:**

In this study we aimed to evaluate the long-term outcome of diaphragmatic plication for symptomatic unilateral diaphragm paralysis.

**Methods:**

Thirteen patients who underwent unilateral diaphragmatic plication (2 patients had right, 11 left plication) between January 2003 and December 2006 were evaluated. One patient died postoperatively due to sepsis. The remaining 12 patients [9 males, 3 females; mean age 60 (36-66) years] were reevaluated with chest radiography, flouroscopy or ultrasonography, pulmonary function tests, computed tomography (CT) or magnetic resonance imaging (MRI), and the MRC/ATS dyspnea score at an average of 5.4 (4-7) years after diaphragmatic plication.

**Results:**

The etiology of paralysis was trauma (9 patients), cardiac by pass surgery (3 patients), and idiopathic (1 patient). The principle symptom was progressive dyspnea with a mean duration of 32.9 (22-60) months before surgery. All patients had an elevated hemidiaphragm and paradoxical movement radiologically prior to surgery. There were partial atelectasis and reccurent infection of the lower lobe in the affected side on CT in 9 patients. Atelectasis was completely improved in 9 patients after plication. Preoperative spirometry showed a clear restrictive pattern. Mean preoperative FVC was 56.7 ± 11.6% and FEV1 65.3 ± 8.7%. FVC and FEV1 improved by 43.6 ± 30.6% (p < 0.001) and 27.3 ± 10.9% (p < 0.001) at late follow-up. MRC/ATS dyspnea scores improved 3 points in 11 patients and 1 point in 1 patient at long-term (p < 0.0001). Eight patients had returned to work at 3 months after surgery.

**Conclusions:**

Diaphragmatic plication for unilateral diaphragm paralysis decreases lung compression, ensures remission of symptoms, and improves quality of life in long-term period.

## Background

Acquired diaphragm paralysis is characterized by the loss of muscle contractility that leads to progressive muscular atrophy and distension of the dome [1]. Diaphragm paralysis may deteoriate the function and efficiency of respiration. It may cause paradoxical motion of the affected diaphragm, atelectasis, and contralateral mediastinal shift. These changes can lead to chronic and progressive dyspnea particularly in adults [1]. Acquired diaphragm paralysis may be caused by trauma, cardiothoracic surgery, infection (e.g. herpes zoster, influenza) neoplastic diseases, or autoimmune pathologies directly involving the diaphragm or the phrenic nerve [1,2]. The idiopathic form is considered the result of a subclinical viral infection. This form generally affects adults and presents more commonly with unilateral involvement.

Surgical correction of acquired unilateral diaphragm paralysis by plication as described by Wright (1985) and Graham (1990) is indicated in any case where there is evidence of respiratory compromise without resolution of the condition [3,4]. The aim of surgical repair is to place the paralyzed diaphragm in a position of maximum inspiration which relieves compression on the lung parenchyma and allows its re-expansion [1].

The previous studies focused on the natural history and potential for recovery from diaphragmatic paralysis in adults. Potential benefits of diaphragmatic plication in adults is still uncertain, especially in long-term period. There is limited data on the long-term outcome of diaphragmatic plication in adults with unilateral diaphragm paralysis [4-8].

In this study we aimed to evaluate the long-term outcome of diaphragmatic plication in adults with symptomatic unilateral diaphragmatic paralysis for an average of 5 years.

## Methods

### Study population

This was a single-arm, long-term retrospective series study. Thirteen adult patients with symptomatic unilateral diaphragmatic paralysis who underwent diaphragmatic plication between January 2003 and December 2006 in Thoracic Surgery Department of the Siyami Ersek Cardiothoracic Training Hospital were included in the study. Patients with an upper motor neuron disease, malignant etiology, severe chronic obstructive pulmonary disease, bilateral diaphragm paralysis, chronic cardiac insufficiency, and mechanically ventilated patients were excluded from the study.

All patients gave written informed consent before study procedures. This study was approved by our Institutional Ethics Committe of the Siyami Ersek Cardiothoracic Training Hospital and conducted in accordance to the latest version of Helsinki Declaration and local requirements.

### Surgical procedure

Diaphragmatic plication was performed through a posterolateral thoracotomy in the 6th or 7th intercostal space using controlateral single lung ventilation. The hemidiaphragm transsected approximately 5 cm initally to avoid intraabdominal organ injury, then plicated from medial to lateral with a series of six to eight parallel U sutures (2-0 polypropylene) until it became taut and flat. The use of larger sutures was avoided, since in the cases not diagnosed early, the diaphragm becomes very thin, causing ruptures at the suture line and preventing the tightening of the diaphragm. Pleural space was drained using single chest tube. Pain control was achieved with a thoracic epidural catheter using 0.5% bubivacaine for 48 hours. Patients were discharged 24 hours after their chest tubes were removed.

### Study procedures

All patients received a standardized evaluation before plication operation that included medical history, physical examination, chest X-ray, flouroscopy or ultrasonography and thorax spiral computed tomography (CT) or magnetic resonance imaging (MRI), pulmonary function tests [forced vital capacity (FVC) and forced expiratory volume in 1 s (FEV1)], and assessment of dyspnea score using Medical Research Council (MRC)/American Thoracic Society (ATS) dyspnea grading system (Table 1) [9]. Patients were reevaluated at postoperative long-term period at an average of 5.4 (4-7) years after diaphragmatic plication. This evaluation included chest X-ray, flouroscopy or ultrasonography, thorax Spiral CT, pulmonary function tests, assessment of the MRC/ATS dyspnea score, and their ability to work.

**Table 1:** The Medical Research Council/American Thoracic Society Dyspnea Grading Method [9]

Grade	Severity	Explanation
Grade 0	None	No trouble with breathing except with strenuous exercise
Grade 1	Mild	Trouble with shortness of breath when hurrying on level or walking up a slight hill
Grade 2	Moderate	Walks slower than people of same age on the level or has to stop for breath walking at own pace on the level
Grade 3	Severe	I stop for breath after walking 100 yards or after a few minutes on the level.
Grade 4	Very severe	Too breathless to leave the house or breathless when dressing or undressing

### Statistical analysis

Study data was summarized using descriptive statistics (number, mean, range, and standard deviation). Wilcoxon signed rank test was used to compare categorical variables. Continuous variables were compared by Student's paired t-test. All tests were two-sided and statistical significance was set at p < 0.05.

## Results

### Patients and preoperative findings

Among 13 patients included in the study, one died in postoperative period due to ventilatory dependency pneumonia and sepsis. This patient had moderate chronic obstructive pulmonary disease (FEV1 = 65% of predicted value) and body mass index was 30 m^2^/kg. The remaining 12 patients [9 males, 3 females; mean age 60 (36-66) years] were followed for long-term after diaphragmatic plication.

**Figure 1:**
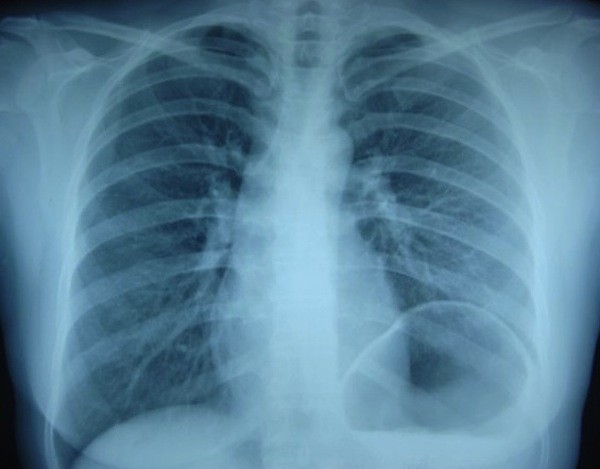
**Preoperative chest X-ray of a 45-year-old female patient with diabetes who had dyspnea for 22 months shows that left diaphragm ascended up to infrahiler level**.

**Figure 2:**
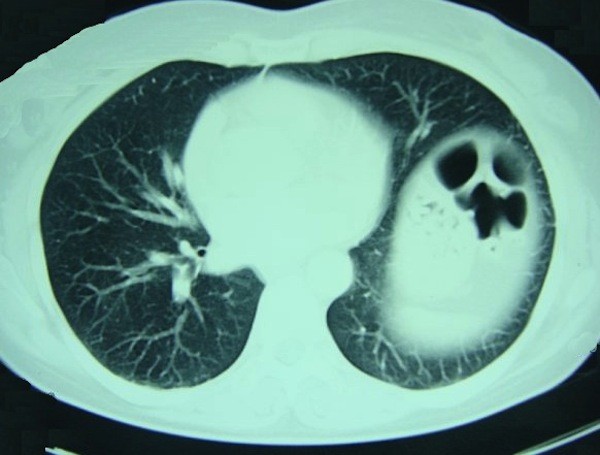
**Spiral CT of the patient in Fig. 1 shows the atelectasis in left lower lobe, and relocation and retraction of mesenteric adipose tissue and colon loops towards diaphragm**.

Patients' demographic and clinical characteristics are displayed in Table 2. The etiology of paralysis was trauma (9 patients), cardiac by pass surgery (3 patients), and idiopathic (1 patient). The principle symptom was progressive dyspnea on exertion with a mean duration of 32.9 (22-60) months before surgery. In addition to dyspnea, 9 patients had respiratory and digestive symptoms such as abdominal discomfort. All patients had an elevated hemidiaphragm in chest X-ray and CT or MRI (Figure 1) and paradoxical movement in ultrasound or flouroscopy and evaluation prior to surgery. There were partial atelectasis and reccurent infection of the lower lobe in the affected side on CT in 9 patients (Figure 2).

**Table 2:** Characteristics of surgically plicated patients (n = 13)

Variable	Result
Age [mean (range)]	60 (36-66) years
Male/female (n)	9/4
Progressive dyspnea (n)	13
Respiratory and digestive symptoms (n)	9
Mean duration of symptom [mean (range)]	32.9 (22-60) months
Etiology (n)	
Idiopathic	1
Cardiac by pass surgery	3
Trauma	9
Operation side (n)	
Right	2
Left	11

### Postoperative findings

Eleven patients including the patient who died in postoperative period had left, and 2 patients had right diaphragmatic plication. Mean lenght of hospital stay was 7 days. Two patients (15.3%) experienced a superficial wound infection. None of the patients died at long-term follow-up.

#### Radiological findings

**Figure 3:**
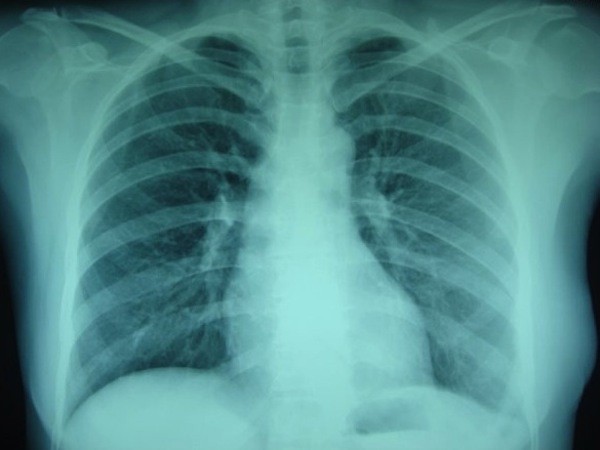
**Chest X-ray of the patient in Fig. 1 at the end of postoperative 3rd year shows that left diaphragm is in normal position and lung is fully expanded**.

**Figure 4:**
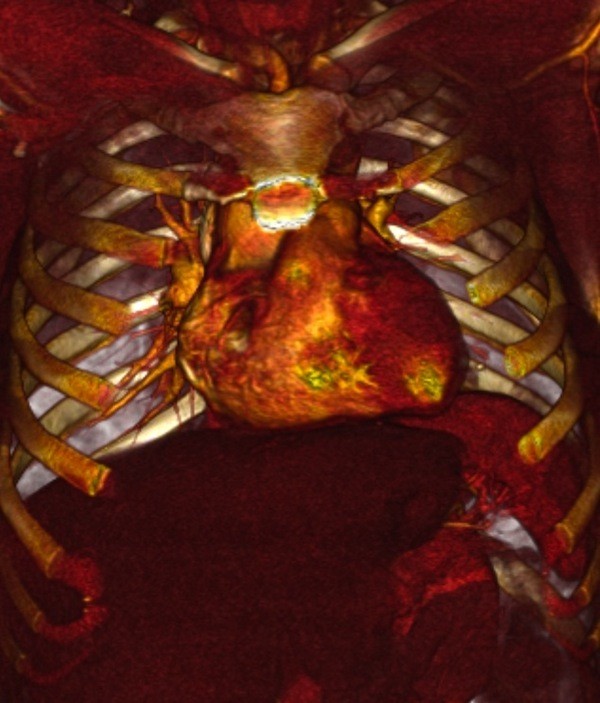
**Three-dimensional multislice reconstruction of the patient in Fig. 1 at the end of postoperative 3rd year**. Plicated left diaphragm is entirely in normal position.

In eleven patients, position of the diaphragm was normal after plication, but the diaphragm was elevated without symptoms in one patient at the end of postoperative 12th month. Flouroscopy showed that surgically plicated diaphragm was immobile and still elevated without any symptom, and there was no paradoxical motion. Atelectasis, which was found in 9 patients preoperatively, completely improved in X-ray (Figure 3) and CT scan after plication (Figure 4).

#### Pulmonary function tests

Preoperative pulmonary function tests showed a clear restrictive patern. Mean preoperative FVC was 56.7 ± 11.6% and FEV1 65.3 ± 8.7% in spirometry. FVC and FEV1 improved by 43.6 ± 30.6% (p < 0.001) and 27.3 ± 10.9% (p < 0.001) at late follow-up (Table 3).

**Table 3:** Spirometry results before and after plication at long-term follow-up

	FVC (%)			FEV1 (%)		
		
Patient no.	Before plication	After plication	Improvement (% change)	Before plication	After plication	Improvement (% change)
1	50.0	79.0	58.0	61.0	72.0	18.0
2	57.0	86.5	51.8	71.0	86.0	21.1
3	50.8	80.5	58.5	62.8	78.8	25.5
4	67.0	104.0	55.2	69.0	105.0	52.2
5	76.0	94.0	23.7	88.7	99.4	12.1
6	76.0	70.0	-7.9	60.2	84.0	39.5
7	47.8	72.5	51.7	58.0	76.5	31.9
8	50.0	60.7	21.4	57.0	76.0	33.3
9	44.0	77.4	75.9	64.0	80.0	25.0
10	59.0	85.0	44.1	69.2	85.5	23.6
11	61.2	58.3	-4.7	58.0	67.7	16.7
12	41.0	80.0	95.1	64.3	82.4	28.1

**Total**	56.7 ± 11.6	79.0 ± 12.9	43.6 ± 30.6*	65.3 ± 8.7	82.8 ± 10.6	27.3 ± 10.9*

*p < 0.001, Student's paired t-test.

#### MRC/ATS dyspnea score

Preoperative MRC/ATS dyspnea score improved from 3 to 0 (3 points) for 11 patients and from 4 to 3 (1 point) in 1 patient at long-term follow-up after plication (p < 0.0001) (Table 4).

**Table 4:** Dyspnea scores before and after plication at long-term period [n (%)]

Dyspnea score before plication			Dyspnea score after plication		
	
0	3	4	0	3	4
-	11 (91.7%)	-	11 (91.7%)	-	-
-	-	1 (8.3%)	-	1 (8.3%)	-

p < 0.0001, Wilcoxon signed rank test.

#### Working history

Eight patients who had left their jobs because of dyspnea had returned to work within 6 months after surgery. The other 4 patients were retired. None of the patients treated with subsequent hospital admission related to pulmonary or digestive complaints and required re-plication.

## Discussion

In this long-term follow-up study, we evaluated an average of 5.4 (4-7) years outcome of diaphragmatic plication in adults with symptomatic unilateral diaphragmatic paralysis. We found that diaphragmatic plication for unilateral diaphragm paralysis reexpands the atelectatic lung, improves respiratory and digestive symptoms, and quality of life in long-term period.

Symptomatic unilateral diaphragmatic paralysis in adult patients is an uncommon but severely disabling clinical problem. The diagnosis of diaphragm paralysis is suggested when the chest X-ray shows a raised diaphgram and is confirmed by fluoroscopy, ultrasonography, Spiral CT, thorax MRI, and most definitively by electromyogram (EMG) stimulation. For differantial diagnosis, spiral CT is used to eliminate particularly thorax malignancies and fiberoptic bronchoscopy is used to define endobronchial patologies due to atelectasis. Particularly multislice CT is a valuable tool for evaluating subdiaphragmatic area, and diaphragm rupture and/or herniation associated with postraumatic diaphragm paralysis [10]. The diagnosis of unilateral diaphragm paralysis may be missed in older patients and postoperative cases. Moreover, the diagnosis is often delayed, unless it follows trauma or cardiothoracic surgery. Nowadays, ultrasound evaluation of diaphragm function is a sensitive, safe, and non-invasive method without radiation exposure and has replaced the use of radioscopy and EMG [11]. The etiology of diaphragm paralysis is usually defined based on the history and previous chest X-ray of the patients.

Careful evaluation of the disease is obligatory prior to surgical correction to differantiate other possible reasons that may lead to respiratory symptoms. Following diagnosis of diaphragm paralysis, surgical treatment is indicated after excluding paranchymal lung disease, chronic heart failure, and neoplastic etiology; and if pulmonary symptoms still persist in spite of treatment of lung infection, physical therapy, and body weight control. Patients should be selected properly for plication surgery to prevent unnecessary operations. Exertional dyspnea severe enough to impair simple daily activity is the most common indication for surgery.(1) However, timing of surgery is still debated. Some authors recommend plication after a period of 3-6 months [1], while others recommend a longer waiting period anticipating the potential spontaneous recovery especially in diaphragm paralysis due to cardiac surgery [12]. Summerhill et al. reported that 11 of 16 patients (69%) functionally recovered from diaphragmatic paralysis and the time for spontaneous recovery ranged from 5 to 25 months (mean 14.9 ± 6.1 months) [11]. Mouroux et al. suggested to wait 18-24 months before the plication surgery for diaphragm paralysis and eventration which is not an objective criteria [13].

The mean time to plication was 32.9 months in our series. This relatively long duration was due to the late diagnosis and late referral of most patients to our clinic rather than long waiting period for surgery.

According to our clinical experience, the waiting period should be at least 12 months depending on the etiology of paralysis.

Plication through standard thoracotomy is the most frequently used surgical technique in diaphragm paralysis. It carries low morbidity and no mortality. Graham et al. treated 17 patients using thoracotomy, and showed that functional improvement was present even at long-term follow-up [4]. Higgs et al. also reported that diaphragmatic plication is an effective treatment for long-term in unilateral diaphragmatic paralysis and showed improvement of spirometry findings at long-term period up to 14 years [5]. Similar results were also reported by Ribet and Linder [6].

The surgical technique preferred in the current study has several advantages. The paralyzed diaphragm is almost always thin, thus it's difficult to avoid injury of abdominal organs just below this thin structure. This surgical technique also gives extratightness and tense to diaphragm by strongly suturing the lowest border of flaccid diaphragm. The standard thoracotomy enables the surgeon to control the diaphragm completely by touching and feeling. Following the incision of the diaphragm and the examination of the underlying organs, the suturing procedure becomes easier with a tightened diaphragm. Strong and tense plication of paralyzed diaphragm is the most important factor for providing favorable long-term surgical outcome. Our experience showed that the only limitation of this technique is long duration of serosanguineous drainage and removal of chest tube at day 3 (2-9) on average. This situation may be due to trauma caused by incision of diaphragm and impaired lymphatic circulation. The incision area of diaphragm should be avascular with no neurons, which may be easily recognized with thinest atrophic structure.

Diaphragmatic plication by video-asissted thoracoscopic surgery (VATS) has been reported by Freeman et al. in a study that showed that all patients who underwent plication of hemidiaphragm through VATS improved in dyspnea and spirometric values at long-term period [7]. However, there is still limited data on the advantages and disadvantages of VATS technique. In the present study, we did not perform plication with VATS. Our recent experience with VATS indicated the difficulty of obtaining a sufficiently tense diaphragm with VATS technique. On the other hand, diaphragm must not be over-tightened because that will restrain the lower chest wall from expanding to prevent limiting inspiration.

The incidence of phrenic nerve dysfunction in adults after coronary artery by pass grafting reported to be 10% to 60% [14-16]. Katz et al. showed that 80% of patients spontaneously recovered in 1 year [17]. However, Kuniyoshi et al. suggested that one of the indications of plication for patients with diaphragm paralysis due to coronary artery by pass surgery is difficult to wean from mechanical ventilation [12]. Kuniyoshi et al. also reported that plication is an effective and safe technique for diaphragm paralysis due to open cardiac surgery in adults as in children [12]. In our study, plication was performed in 3 patients with diaphragm paralysis due to coronary artery by pass surgery. In these 3 patients, the internal mammary artery had been used for by pass surgery and duration of dyspnea was over 15 months.

Diaphragmatic paralysis after coronary artery by pass grafting in adult patients is commonly attributed to topical cooling [16,17]. However, topical cooling is not currently used, which decreased the frequency of diaphragm paralysis. One of the possible causes of diaphragm paralysis after coronary artery by pass grafting is harvest of internal mammary artery. It was shown that phrenic nerve crosses over internal mammary artery in anterior thoracic wall in 54% of patients and in posterior thoracic wall in 14% of patients [18]. Furthermore, pericardiophrenic artery originates from internal mammary artery in 89% of cases [19,20]. In case of thermal injury of internal mammary artery by electroknife, phrenic nerve may become ischemic. In addition to surgical technique, diabetes and older age have been considered as potential risk factors for diaphragm paralysis [20,21].

In the present study, MRC/ATS dyspnea scale was used to evaluate the subjective effect of diaphragm plication on symptoms. Dyspnea score was first used for assessment of shortness of breath by Higgs et al. MRC and ATS dyspnea scoring systems are currently the most commonly used dyspnea evaluation tools [5]. These systems are based on the assessment of apparent dyspnea by 5 different severity statements. While Simansky et al. used ATS dyspnea scoring system, Freeman et al. used MRC system; and both studies reported that dyspnea was improved in long-term after plication surgery and majority of patients returned to their work [22,7]. Versteegh et al. performed lateral thoracotomy in 15 patients with unilateral diaphragm paralysis and found that all patients showed subjective and objective improvement [22]. However, they used baseline dyspnea index in preoperative period and transition dyspnea in postoperative period as described by Witek and Mahler [23]. These indexes evaluates the magnitude of functional impairement for task provoking dyspnea and the magnitude of the effort associated with that task. But these indexes are not easy to understand and the application of them is more difficult, thus they are not practical to use in routine.

One patient in our series died in postoperative 60th day due to sepsis and multiorgan failure as a result of ventilatory pneumonia after prolonged entubation. This patient had moderate chronic obstructive lung disease, and body mass index was 30 m^2^/kg. Diaphragm paralysis patients with chronic obstructive lung disease and obesity have high risk for morbidity and mortality. This experience has taught us that plication must not be applied in the patients with an ejection fraction below 40, in the patients with moderate to severe chronic obstructive lung disease and to the patients with a body-mass index of 30 m^2^/kg or above. Even though plication was performed in these patients, long-term intense bronchodilator treatment and respiration physiotherapy should be applied, and patients should be encouraged to lose weight. Versteegh et al. reported preoperative 3 deaths among series of 22 patients who underwent plication. Deaths were due to heart attack, massive pulmonary embolism, and renal failure and right heart failure [8]. Pathak and Page reported splenic injury due to plication for which they suggested the incision of diaphragm to control the underneath tissues [24]. Phadnis et al. reported abdominal compartment syndrome after right plication surgery [25]. They speculate that their patient had abdominal compartment syndrome develop as a consequence of downward hepatic shift and reduced intra-abdominal volume. Mortality related to surgical procedure has not yet been reported.

## Conclusion

As a conclusion, diaphragm paralysis patients showed both objective and subjective improvement in long-term period after plication. Hence, it ensures remission of symptoms, and improves quality of life in long-term period.
